# In-Growing Toe-Nail

**Published:** 1897-02-13

**Authors:** W. McAdam Eccles

**Affiliations:** Assistant Surgeon West London Hospital, City of London Truss Society, Surgeon St. Marylebone General Dispensary, &c.


### I.—IN-GROWING TOE-NAIL.

By W. McAdam Eccles, M.S. (Lond.), F.R.C.S. (Eng.), Assistant Surgeon West London Hospital, City of London Truss Society, Surgeon St. Marylebone General Dispensary, &c.

Quite a number of common, and in some senses, minor ailments are but little studied, and are perhaps, therefore, apt to be somewhat crudely treated. It is my intention to bring forward two of these, and to seek to give some practical hints which I have gathered from the observation of a large series of cases.

### In-Growing Toe-Nail.

This is a very frequent affection, and one which I venture to say has an entirely erroneous name attached to it. It would be supposed from the usual appellation of "in-growing toe-nail" that the nail of the great toe, which is the particular one that is almost invariably the site of the trouble, had a tendency to grow of its own accord into the soft tissues by its side. It might be almost said that it deliberately and with malice aforethought seeks to invade the unoffending sensitive structures lying so close to it. This view of the condition is a distinct calumny. The nail does not behave in so ruthless a manner. It has also been further asserted that if the nail is not at fault then the ailment must be termed an overgrowth of the soft parts. But this again is, as it were, adding insult to injury, for the soft tissues have no more tendency to overshadow the nail than the latter has to thrust itself into the former.

A glance at the essential cause of the affection will surely throw light as to the true way in which such a distressing complaint is brought about, and thus give a clue as to its proper nomenclature. The toes, which in a state of nature are not given to crowd on one another, are unreasonably forced into a more or less unyielding covering. This so confines them, and further, in far too many cases, produces pressure upon them, that the soft tissues give under the force, and are pushed, whether they will or no, against and over the nail itself. Here, then, is a clear case of "over-pushed soft parts," and I think such is the proper term to employ in designating the trouble. This abnormal state of affairs leads to intense suffering on the part of the one who has acquired it. The highly sensitive tissue is terribly injured by the contact with the sharp edge of the nail, and in many cases an actual wound is thereby produced. When this happens it allows the introduction of septic micro-organisms, and therefore active inflammation follows, resulting often in the formation of a mass of granulation tissue, which still further adds to the mass lying upon and over the nail. A most marked fœtor from decomposition is an almost invariable accompaniment.

Victims of this apparently trivial affection are seriously inconvenienced, and in many cases dread to seek surgical assistance from an inherent horror of the idea of avulsion of their toe-nail—a treatment which many think is the sole one for their trouble. Perhaps but few of such simple lesions have had their treatment founded upon more unsound pathology than this one.

The usual designation has led many practitioners to believe that the prime factor in the mischief was in the nail itself, and therefore they have sought to annihilate its influence for the bad by effecting its entire removal. This operation, besides the all-important fact of its being entirely unnecessary, has the distinct disadvantages of being extremely painful, and therefore requiring in almost every instance a general anæsthetic, and in addition of leaving a wound which takes a considerable time to heal, thus needlessly incapacitating a patient from his ordinary duties. The less severe measure of scraping or rasping the middle part of the nail, so that it becomes thin and thus will yield to the lateral pressure upon it, is sometimes advocated. This treatment again, however, is based upon the same untrue ideas of the causes of the affection, seeing that no attempt is made in most cases to remove the pressure itself. Another method which is employed is that of cutting away the overlying soft tissues, and thus obtaining cicatrisation—often after a considerable period of time—which prevents further development of the condition. But it is not the soft parts which are at fault, in the sense that they have grown over the nail, and must therefore be removed; in fact, to my mind this is also an unnecessary mutilation. What then should be the rational line of treatment with the greatest hope of success in these cases?

First, and if this primary essential is not obtained all else will fail, the pressure which has been acting so banefully must be for once and all entirely removed. In other words, boots which are properly fashioned and fitted must at once be obtained. During the actual treatment of the condition of the toe, I often advise that only quite yielding material, such as canvas shoes, should cover the affected area.

Secondly, an attempt must be made to render the part aseptic. This will be best done by soaking the whole foot in a hot solution of 1-4000 biniodide of mercury, after having previously washed it well with soap and water.

Thirdly, a small piece of antiseptic gauze should be gently and very gradually, but at the same time thoroughly, insinuated between the nail and the overlying granulation tissue and soft parts. This is an extremely painful operation if at all roughly done, and once a patient has endured any harsh manipulation he will greatly dread a repetition of it. However, after a thorough soaking in the manner described, and with great care in the method of introducing the gauze, very little, if any, pain need be caused.

The small piece of gauze will retain itself in position, but should be removed the next day. It will now be found that the tissues are markedly separated from the nail, and this latter can at this stage be easily cut away where its outer edge is pressing against the soft parts. Another prolonged application of the antiseptic solution should be carried out, and the gauze again inserted. The patient will soon learn to do this for himself, and it is astonishing how quickly all the tenderness and pain vanishes, and how rapidly the granulation tissue shrinks up and the purulent fœtid discharges disappear. Within a week the condition is practically cured, and it only remains that a strip of gauze should be inserted each morning for about a month or six weeks, and that properly made boots be continued to be worn, in order that permanent relief may be secured.

